# Retina-Targeted Delivery of 17β-Estradiol by the Topically Applied DHED Prodrug

**DOI:** 10.3390/pharmaceutics12050456

**Published:** 2020-05-16

**Authors:** Katalin Prokai-Tatrai, Vien Nguyen, Daniel L. De La Cruz, Rebecca Guerra, Khadiza Zaman, Fatima Rahlouni, Laszlo Prokai

**Affiliations:** Department of Pharmacology and Neuroscience, University of North Texas Health Science Center, Fort Worth, TX 76107, USA; Vien.Nguyen@unthsc.edu (V.N.); Daniel.DeLaCruz@my.unthsc.edu (D.L.D.L.C.); rebecca.guerra@unthsc.edu (R.G.); Khadiza.Zaman@unthsc.edu (K.Z.); Fatima.Rahlouni@my.unthsc.edu (F.R.); Laszlo.Prokai@unthsc.edu (L.P.)

**Keywords:** 17β-estradiol, 10β,17β-dihydroxyestra-1,4-dien-3-one, DHED, eye drops, glaucoma, ocular neuroprotection, prodrug, rabbit, retina-targeted drug delivery, visual function

## Abstract

The purpose of this study was to explore retina-targeted delivery of 17β-estradiol (E2), a powerful neuroprotectant, by its bioprecursor prodrug 10β,17β-dihydroxyestra-1,4-dien-3-one (DHED) administered as eye drops in animal models. Compared to the parent hormone, DHED displayed increased transcorneal flux ex vivo both with and without the presence of 2-hydroxypropyl-β-cyclodextrin used as a penetration-enhancing excipient in rat, rabbit, and pig. In vitro, the prodrug also showed facile bioactivation to E2 in the retina but not in the cornea. After topical administration to rats and rabbits, peak DHED-derived E2 concentrations reached 13 ± 5 ng/g and 18 ± 7 ng/g in the retina of female rats and rabbits, respectively. However, the prodrug remained inert in the rest of the body and, therefore, did not cause increase in circulating hormone concentration, as well as wet uterine and anterior pituitary weights as typical markers of E2′s endocrine impact. Altogether, our studies presented here have demonstrated the premise of topical retina-selective estrogen therapy by the DHED prodrug approach for the first time and provide compelling support for further investigation into the full potential of DHED for an efficacious and safe ocular neurotherapy.

## 1. Introduction

The beneficial effects of endogenous or exogenously applied 17β-estradiol (E2) for eye health have been shown by basic science studies [1,2,3,4,5] and epidemiological observations [6,7,8]. Our interest in this regard stems from an unmet medical need for ocular neuroprotection [3,9]. This condition requires chronic treatments, and therefore, considerations for therapeutic safety are especially important [10]. It has been generally accepted that the broad-spectrum neuroprotection exerted by E2 is owing to a synergistic combination of its non-genomic and genomic actions [11,12]. The latter involves the nuclear estrogen receptors (ERα and ERβ) expressed throughout the eye including the retina [13]. The presence of the phenolic A-ring in the steroid structure makes E2 unique among neurosteroids. This feature is associated with one of the best-known non-genomic effects of E2, which is its ability to act as a direct free-radical scavenging phenolic antioxidant [14,15]. This antioxidant capacity is a critical contributor to the amelioration of oxidative stress heavily implicated in the initiation and/or progression of neurodegeneration [16].

Previously, we have shown that topically applied E2 provided protection for the retinal ganglion cell (RGC) layers in an established animal model of glaucoma [3]. In this paradigm, the intraocular pressure (IOP) is increased by episcleral vein injection of hypertonic saline [17]. RGCs are neurons that connect the eyes to the brain. In glaucoma, RGC and their axons forming the optic nerve are gradually destroyed leading to vision loss even when the IOP is controlled [18]. In our study [3], approximately 25% elevation of IOP in the ipsilateral eye produced a significant RGC death with the expected loss of visual acuity in ovariectomized (OVX) rats having no significant circulating E2 to confound results. Once-daily (q.d.) E2 eye drop treatment for three weeks did, however, result in a large E2 concentration in the retina and provided profound neuroprotection by preventing the deterioration of visual acuity. Through mass spectrometry-based proteomics, we have also identified several protein targets in numerous protein networks implicated in retina health upon this topical E2 treatment schedule [19]. On the other hand, treatment with eye drops containing the hormone also produced high circulating E2 level and, therefore, triggered unwanted side-effects such as adverse impact on the uterus of experimental animals [3].

Increased blood E2 content and associated undesirable peripheral hormonal liability upon systemic E2 administrations for the treatment of the central nervous system (CNS) are unavoidable with any currently known non-invasive method of drug delivery. This is an obstacle toward realizing safe E2-based neurotherapy. High blood E2 levels have been associated with an increased risk of developing certain types of cancers, as well as cardiovascular side-effects [20]. Also, feminization by the hormone further hinders the utilization of its many beneficial effects in men [21]. Altogether, these caveats justify efforts to localize the therapeutic benefits of E2 to the site of action. In essence, targeted drug delivery is needed to ensure therapeutic safety and efficacy to prevent, treat and/or halt neurodegeneration or to alleviate estrogen-responsive maladies requiring long-term pharmacological interventions.

**Figure 1:**
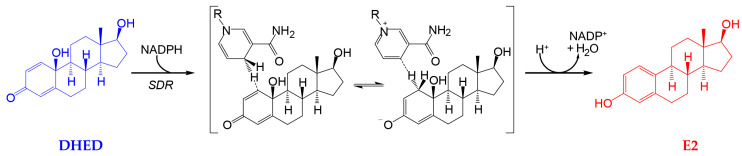
10β,17β-dihydroxyestra-1,4-dien-3-one (DHED) is a bioprecursor prodrug converted to 17β-estradiol (E2) in neuronal environments through NADPH-dependent enzymatic reduction [22,23]. SDR, short chain dehydrogenase/reductase; only the 1,4-dihydronicotinamide and the corresponding pyridinium moieties are displayed for NADPH and nicotinamide adenine dinucleotide phosphate (NADP^+^), respectively, with the rest of the structure not participating in the reaction indicated by R.

Recently, we have identified a first-in-class bioprecursor prodrug resulting in the highly localized and rapid formation of E2 in the brain upon systemic administrations [9,22,23]. As shown in Figure 1, this small-molecule bioprecursor prodrug (10β,17β-dihydroxyestra-1,4-dien-3-one, DHED) selectively metabolizes in the brain to E2 by an enzyme-catalyzed process relying on the reduced form of nicotinamide adenine dinucleotide phosphate (NADPH) as a cosubstrate. The resultant site-specific E2 delivery allowed for concomitant therapeutic benefits in several animal models of brain maladies including neurodegeneration such as Alzheimer’s disease [22,23,24,25,26]. Importantly, we have also shown that DHED remained inert in the rest of the body independently of the routes of prodrug administration; thus, it did not produce an increase in circulating E2 and, therefore, associated adverse peripheral effects. Encouraged by these findings, we probed whether topical DHED in the form of eye drops produces E2 locally in the retina without systemic hormonal liabilities. The retina is part of the CNS, and it is also impaired in patients with neurodegeneration in the brain [27]. Therefore, potential retina-selective delivery of E2 by DHED is a plausible hypothesis from our previous work [10,19,22].

While topical application of ophthalmic agents (especially for the treatment of the posterior segment of the eye) have numerous limitations including low ocular bioavailability and systemic drug absorption leading to unwanted adverse effects [28], patients prefer this route of administration to invasive injections [29]. DHED also appears ideal in size and lipophilicity for the transcorneal route of drug absorption [28,29]. Along with comparative characterization of corneal permeability and in vitro metabolism in ocular tissues, we also provide in vivo evidence here for the retina-selective formation of E2 from this bioprecursor prodrug when it is applied topically in eye drops to rats and rabbits.

## 2. Materials and Methods

### 2.1. Chemicals and Reagents

DHED, 10β,17β-dihydroxyestra-1,4-dien-3-one-16,16,17-d_3_ (d_3_-DHED) and 10β,17β-dihydroxy- estra-1,4-dien-3-one-2,4,16,16,17-d_5_ (d_5_-DHED) were synthesized in our laboratory as reported before [22,30]. 17β-Estradiol-16,16,17-d_3_ (d_3_-E2) and 17β-estradiol-2,4,16,16,17-d_5_ (d_5_-E2) with 98% isotope purity were purchased from CDN Isotopes (Pointe-Claire, QC, Canada). 17β-Estradiol-13,14,15,16,17,18-^13^C_6_ (^13^C_6_-E2) with 99% isotope purity was obtained from Cambridge Isotope Laboratories (Andover, MA, USA). Water, acetonitrile, acetic acid and formic acid were Optima^®^ LC/MS grade, and supplied by Thermo Fisher Scientific (Waltham, MA, USA). All other chemicals were obtained from MilliporeSigma (St. Louis, MO, USA).

### 2.2. Animals

All animal procedures were carried out in accordance with the ARVO resolution for the Use of Animals in Ophthalmic and Vision Research. All protocols before the initiation of the studies were approved by the Institutional Animal Care and Use Committee at the University of North Texas Health Science Center (approval numbers: 2018-0028, 2019-0033 and 2019-0016). OVX Brown Norway rats (150–200 g) were purchased from Charles Rivers Laboratories (Wilmington, DE, USA). The ovariectomies were performed by the supplier. Female New Zealand white rabbits weighing 1.0–1.5 kg were also supplied by Charles Rivers Laboratories (Wilmington, DE, USA). Animals had full access to standard diet and water.

### 2.3. Corneal Permeability and In Vitro Metabolism Studies

Experiments were carried out following a published protocol [31] in triplicate using jacketed Franz diffusion cells (PermeGear, Hellertown, PA, USA) with a 9 mm i.d. × 18 mm o.d. spherical joint for pig cornea, 3 mm i.d. × 12 mm o.d. spherical joint for rat cornea, and 5 mm i.d. × 12 mm o.d. spherical joint for rabbit cornea. Receptor volumes were 5 mL. Whole eye globes were purchased from Sierra for Medical Science (Whittier, CA, USA), shipped overnight in PBS over wet ice. Corneas were then immediately excised as used. Test agents (DHED and d_3_-E2) in the donor chamber were in either saline or 20% *w*/*v* (2-hydroxypropyl)-β-cyclodextrin (HPβCD) in saline. At each time point (0, 30, 45, 60, 90, and 120 min), 200 μL of perfusate was removed from the sampling port of the receiving chamber for subsequent drug quantitation and immediately replaced with the same volume of fresh 35 °C medium. Then, the perfusates were spiked with internal standards (ISs: ^13^C_6_-E2 for d_3_-E2 and E2 measurements, and d_5_-DHED for DHED quantitation, respectively), diluted with 1 mL water, and extracted with 2 mL of *tert*-butyl methyl ether. The organic layers obtained from the liquid−liquid extraction were removed, transferred and split equally, and then dried to yield samples for subsequent analyses by liquid chromatography–tandem mass spectrometry (LC-MS/MS). The dried residue from one aliquot was reconstituted in 30 μL of 50% (*v*/*v*) acetonitrile containing 1% (*v*/*v*) acetic acid and assayed for DHED content using atmospheric-pressure chemical ionization (APCI) LC-MS/MS [22]. The residue from the other aliquot was derivatized with dansyl (Dns) chloride and analyzed for E2 and d_3_-E2 by electrospray ionization (ESI) LC-MS/MS, as reported before [32]. The steady-state flux (J_ss_, in pmol·cm^−2^·h^−1^) for each concentration of the analytes was calculated as J_ss_ = (dQ/dt) × A^−1^, where A is the exposed substrate surface area determined by the i.d. (in cm) of the Franz diffusion chamber and dQ/dt denotes the steady-state slopes of linear plots of concentration in the receiving chamber (Q in pmol) versus time (t in h). In vitro metabolism studies were done according to our previously published protocol using fresh tissue homogenates (in pH 7.4 phosphate buffer) [22,33,34]. Briefly, freshly harvested tissues were immediately homogenized, warmed up at 35 °C for 5 min, then spiked with DHED (100 nmol/mL). Aliquots (20 µL) were taken at 2.5, 5, 15, 30, and 60 min, extracted with 2-volume of ethyl acetate and then analyzed using selected reaction monitoring (SRM) of *m*/*z* 271 [M+H–H_2_O]^+^ → *m*/*z* 161, 175 and 189 for DHED and the SRM of *m*/*z* 255 [M+H−H_2_O]^+^ → *m*/*z* 133, 159, 173 for E2. The parent estrogen was unequivocally identified based on co-elution with an authentic reference compound and by MS/MS. For LC/APCI-MS/MS analysis, 5 cm × 2.1 mm i.d. Discovery HS C18 column (MilliporeSigma, St. Louis, MO, USA) was used, and the mobile phase consisted of 65/34/1 water/acetonitrile/acetic acid at 0.3 mL/min flow rate.

### 2.4. Topical Drug Treatment and Sample Preparation for Drug Quantitation

Solutions for eye drops (0.1% *w*/*v*) in 20% *w*/*v* HPβCD in saline were prepared as reported before [3,19,35]. Animals (*n* = 4 per treatment group) received a single eye drop (10 µL for rats and 40 µL for rabbits) in each eye. Control animals (*n* = 6) received vehicle (20% *w*/*v* HPβCD in saline) eye drops only in both eyes. After predetermined time points, animals were euthanized. Blood was collected by cardiac puncture following euthanasia. Followed by a 10-min perfusion with cold PBS through the left ventricle, the eyes were immediately enucleated. Then, retina and major eye parts were quickly dissected, rinsed with saline, blotted dry, and weighed. Other off-target body parts—uterus and anterior pituitary (AP)—were also collected. Wet uterine and AP weights were also recorded. Retina homogenates (in 600 µL of pH 7.4 phosphate buffer) were prepared, spiked with 100 pg of ^13^C_6_-E2 and 1000 pg of d_5_-DHED as ISs, and extracted with 4-volume of *tert*-butyl methyl ether. The organic layers obtained from the liquid–liquid extractions were removed and aliquoted. One third of the organic layer was transferred to reacti-vial (Supelco, Bellefonte, PA, USA) and evaporated under a nitrogen stream to yield samples for derivatization and subsequent LC-MS/MS analysis [32]. The dansylated samples were centrifuged, transferred to autosampler vials, sealed, and assayed. The other portion of the extract was evaporated separately for DHED quantitation [22]. Sera (100–300 μL) were extracted and processed analogously.

### 2.5. LC-MS Analyses

Identification of E2 formed in vitro from DHED was done by LC–APCI-MS/MS on a quadrupole ion trap instrument (LCQ, Thermo Scientific, San Jose, CA, USA) as described previously [36]. Drug quantitation was based on the principles of isotope dilution using validated assays [32,37]. Samples from the corneal permeability studies were analyzed on a TSQ Quantum Ultra triple-quadrupole mass spectrometer (TSQ, Thermo Electron Corporation, Trace Chemical Analysis, Austin, TX, USA) operated in positive ion mode with either an APCI or a heated ESI (HESI) probe to a Surveyor LC System and operated with Xcalibur (version 2.2, Thermo Fisher Scientific, Waltham, MA, USA) data acquisition software. SRM with unit mass resolution for the precursor and product ions was used for quantification. For APCI analysis of DHED, LC separations were carried out on a Supelco (Bellefonte, PA, USA) Discovery HS C-18 reversed-phase column (50 × 2.1 mm, 5 μm) using isocratic elution with a flow rate of 0.3 mL/min. The eluent system consisted of (A) 1% (*v*/*v*) acetic acid in water and (B) 1% (*v*/*v*) acetic acid in acetonitrile. Isocratic eluent composition was set at 35% (*v*/*v*) solvent B. SRM transitions of *m*/*z* 289 → 123 and 294 → 125 were used for DHED and d_5_-DHED, respectively. For HESI analysis of dansylated estrogens, LC separations were carried out on a Phenomenex (Torrance, CA, USA) Kinetex phenyl-hexyl column (50 × 2.1 mm, 2.6 μm) using gradient elution with a flow rate of 0.4 mL/min. The eluent system consisted of (A) 0.1% (*v*/*v*) formic acid in water and (B) 0.1% (*v*/*v*) formic acid in acetonitrile, as reported before [22]. SRM transitions of *m*/*z* 506 → 171, 509 → 171, and 512 → 171 were set up for Dns-E2, Dns-d_3_-E2, and Dns-^13^C_6_-E2, respectively. Samples from in vivo experiments for E2 quantitation after eye drop treatments were also analyzed on the same MS instrument as above in positive-ion HESI mode. Gradient separations were carried out using a Vanquish ultra-high performic liquid chromatography (UHPLC) system (Thermo Electron Corporation, Trace Chemical Analysis, Austin, TX, USA). A Kinetex phenyl-hexyl UPLC column (100 Å, 50 × 2.1 mm, 1.7 µm) from Phenomenex (Torrance, CA, USA) was used for chromatographic separations at 0.4 mL/min flow rate. SRM was set up analogously as above for samples from the transcorneal permeability studies. Prodrug quantitation from in vivo samples was done analogously to those of ex vivo permeability studies except for utilizing the Vanquish UHPLC system for chromatographic separations. 

### 2.6. Statistical Analyses

Descriptive statistics were calculated for each group and for all outcomes. Transcorneal fluxes were evaluated by analysis of variance (ANOVA) considering 2 × 2 × 3 factorial design [38] with compound (E2 and DHED), formulation (without and with HPβCD), and animal species (rat, rabbit, and pig) as independent variables. Effect sizes were estimated from ANOVA results as the proportion of the total variance that is attributed to the effect or interaction (eta squared, η^2^) [38]. Differences of mean values between experimental groups were assessed by unpaired Student’s *t*-test or with one-way ANOVA followed by post hoc Tukey tests. In all statistical analyses, *p* < 0.05 was considered statistically significant.

## 3. Results

### 3.1. Transcorneal Permeability and In Vitro Metabolic Stability Studies

**Figure 2:**
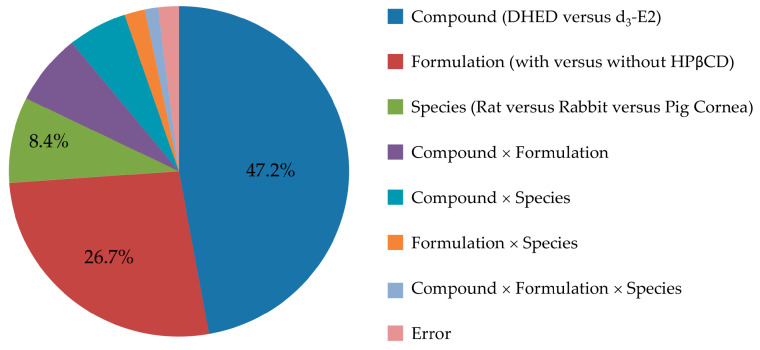
Relative effect sizes (η^2^ × 100) for compound, formulation, choice of animal species and their interactions calculated from the ANOVA of our 2 × 2 × 3 factorial experiment evaluating transcorneal permeabilities (Table 1 and Table S1).

We first surveyed the transcorneal permeability of DHED in relation to the parent E2 as a predictor for the feasibility of topical application. In order to also detect potential E2 formation, if any, from DHED during transport through the cornea our comparative studies between prodrug and parent drug necessitated the use of a stable isotope-labeled E2 rather than E2. Therefore, we selected d_3_-E2 based on considerations for drug quantitation by LC-MS/MS on the principles of isotope dilution and using ^13^C_6_-E2 as IS [32]. Moreover, the study was performed in a comprehensive experimental design considering both a comparison of DHED and E2 and evaluating the effect of a penetration-enhancing excipient (HPβCD) [39] in the ophthalmic vehicle, as well as rat, rabbit and pig as species among the factors. Our results were summarized in Table 1, and effect sizes estimated from ANOVA η^2^ values (Table S1) are displayed in Figure 2.

Another conceptually important finding from this study in the context of retina-selective delivery of E2 by DHED eye drops was that no prodrug bioactivation and, thus, no E2 formation could be detected during corneal transport in the perfusates by LC-MS/MS (Figure S1A). This finding was also rigorously verified by incubating DHED (100 nmol/mL) in cornea homogenate (20% *w*/*v*, pH 7.4 PBS) followed by LC-MS/MS analysis. At the same time, conversion of DHED to E2 proceeded rapidly in the retina with 533 ± 158 nM·min^−1^·mg protein^−1^ (average ± SEM, *n* = 3) initial rate of formation (Figure S1B).

### 3.2. Topical DHED Produces Large Concentration of E2 in the Retina without Peripheral Exposure to the Hormone

**Figure 3:**
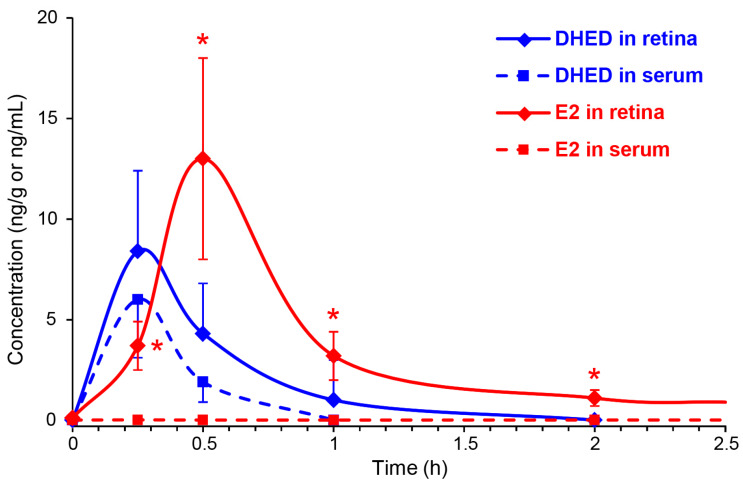
DHED (blue) and E2 (red) concentrations in the retina (solid line) and blood (dashed line) after a single eye drop (10 µL) of 0.1% *w*/*v* DHED into the eyes of ovariectomized (OVX) rats. Data are mean ± SD, *n* = 4. * *p* < 0.05 compared to control (displayed at 0 h).

We instilled a single drop (10 µL) of 0.1% (*w*/*v*) DHED into the eye of OVX Brown-Norway rats, similarly to our earlier studies [3,19,35]. Animals were then sacrificed at 15, 30, 60 and 120 min, as well as 24 h post-dosing. DHED and E2 contents in the blood and retina were determined by LC-MS/MS [32]. Figure 3 shows the time course of DHED in the retina and serum. After 1 h, the presence of the prodrug in these matrices was no longer quantifiable with our LC-MS/MS assay [22]. Concomitant with the appearance of DHED, a significant E2 concentration was also measured in the retina and, importantly, without an increase in circulating E2. The maximum retinal E2 (13 ± 5 ng/g) and DHED (8.4 ± 4 ng/g) concentrations were measured approximately 30 min after eye drop administration. In control animals, we detected 8 ± 3 pg/mL serum E2, and no significant changes in this measure was determined (e.g., 9 ± 4 pg/mL after 30 min and 8 ± 4 pg/mL after 24 h) when DHED eye drops were administered. At 24 h time point past DHED administration, the retina had 425 ± 176 pg/g E2 content.

When the animals received q.d. eye drops for three weeks, similarly to our earlier studies with the parent agent [3], E2 content of the retina was 389 ± 141 pg/g 24 h after the last topical treatment, indicating that the hormone did not accumulate in this eye compartment. As mentioned, previously we have shown that this treatment schedule with the parent E2 eye drops provided therapeutic benefits in an animal model of ocular neurodegenerations [3]. Therefore, it is not surprising that profound neuroprotection could also be seen upon identical treatments with DHED eye drops (Figure S2), as E2 produced locally in the retina from the prodrug protected RGC against IOP-induced damage and, therefore, preserved visual function in the established animal model of glaucoma [17]. However, the significant difference in the outcome between the two treatments (DHED versus E2) was the large increase in circulating E2 after using the hormone itself. Table 2 shows that E2 eye drops produced 453 ± 98 pg/mL serum E2 in sharp contrast with that of DHED eye drops, indicating complete elimination of this unwanted side-effect (9± 4 pg/mL versus 8 ± 3 pg/mL in the control group).

**Figure 4:**
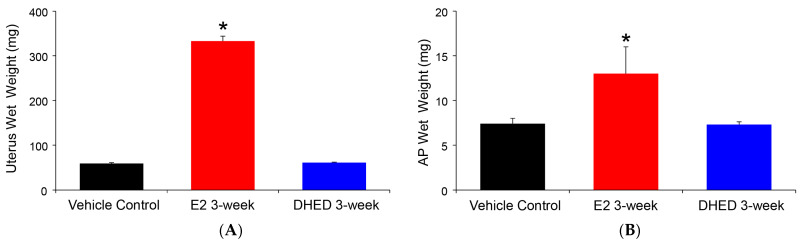
Assessment of peripheral exposure to E2 in ovariectomized (OVX) rats after three-week q.d. DHED or E2 eye drops (10 μL, 0.1% *w*/*v* in 20% *w*/*v* HPβCD in saline solution). Tissues were collected 24 h after the last treatment. Control animals received vehicle eye drops only. (**A**) Wet uterine weights. (**B**) Anterior pituitary (AP) wet weights. Data are given as average ± SD (*n* = 6). * Statistically significant difference to control and DHED treatments, ANOVA followed by Tukey test (*n* = 6, *p* < 0.05).

The beneficial consequence of the site-selective formation of E2 from the topically applied prodrug was the lack of adverse effect on the uterus and AP, as shown in Figure 4. The uterus is extremely sensitive to the presence of circulating E2, and its weight gain due to fluid imbibition is a simple but established measure of peripheral estrogenicity [40]. Similarly, peripheral estrogen-induced AP growth has been well established [41]. Altogether, these complimentary measures are in complete agreement with the lack of increase in circulating E2 after DHED treatments, as measured by our LC-MS/MS assay (Table 2).

**Figure 5:**
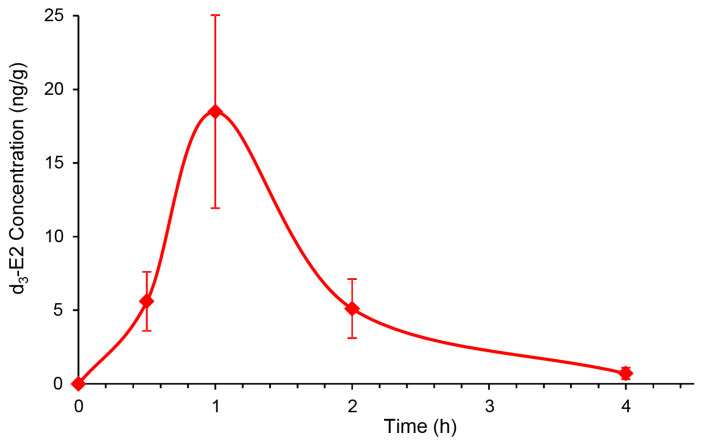
Exploratory survey of the concentration of d_3_-E2 in the female New Zealand white (NZW) rabbit retina after a single eye drop (40 µL) of 0.1% *w*/*v* d_3_-DHED. Data are mean ± SD, *n* = 4.

After we established that topical DHED generates E2 without peripheral hormonal liability in the OVX rat retina (Table 2, Figure 3 and Figure 4), we next explored whether DHED eye drops would reach the back of the eye and metabolize into E2 in the retina of animals whose eyes are more similar in size and anatomy to those of humans. Intact female New Zealand white (NZW) rabbits were selected for this study, as the rabbit model is commonly used in ocular therapy development [42]. To avoid interference with endogenous E2 with that of E2 formed from DHED, we used d_3_-DHED eye drops and, therefore, monitored the appearance of d_3_-E2 in the blood and retina by an LC-MS/MS-based bioassay [32]. We found that, similar to our studies in OVX rats (Figure 3), the maximal steroid concentration in the retina was measured approximately 1 h after dosing (Figure 5), when female rabbits received a single eye drop (40 µL) of 0.1% *w*/*v* prodrug. In the meantime, concentration of d_3_-E2 in the collected serum samples was below 1 pg/mL after the initial slight increase at 15 min time point (18 ± 9 pg/mL). Alongside, the presence of the prodrug in the blood was below the level of quantitation (1 ng/mL) 1 h post-dosing with 8 ± 2 ng/mL at 15 min time point.

## 4. Discussion

This study was designed to explore the utility of topical DHED therapy for retina-selective delivery of E2, a potent ocular neuroprotectant [1,2,3]. Considering eye drops as the most common ophthalmic dosage forms, the evaluation of transcorneal permeability is important even in an early phase of preclinical drug discovery and development. In this regard, our conclusions were drawn from statistical analyses of the results based on a comprehensive study design aimed at assessing not only the comparison of DHED to E2, but also the potential benefits of using a penetration enhancer (HPβCD) [39], as well as the choice of animal species (rat, rabbit and pig) upon measuring steady-state fluxes through isolated corneas in vitro [31]. Overall, all factors (test agent, formulation, and species) and their interactions reached statistical significance upon rigorous statistical analyses (ANOVA, Table S1), thus justifying our experimental design. However, the largest effect sizes were clearly linked to the replacement of the parent estrogen with DHED (η^2^ of 0.472) followed by the use of HPβCD (η^2^ of 0.267). These factors explained >70% of the total variance attributable to the effect on the measured J_ss_ values in our study, as shown in Figure 2.

In view of the unique architecture of the cornea (a lipid-rich epithelium, a lipid-poor soma and a lipid-rich endothelium), a lipophilic-hydrophilic balance in topically applied agents are highly desirable for the transcorneal route [9] that is the major pathway for absorption of drugs having small/medium size (<500 Da) and intermediate lipophilicity [43,44]. Previously, we have reported that the calculated logarithm of the octanol/water partition coefficient (clogP) was 1.67 for DHED that is in sharp contrast with that of highly lipophilic parent E2 (clogP = 4.01) [22]. The practically ideal lipophilicity of DHED in this regard [22,43] projected an excellent corneal absorption for the prodrug in relation to E2. As shown in Table 1, approximately 10-fold higher J_ss_ values were determined for DHED compared to those of the parent compound in all species studied, while saline vehicle (Vehicle A) was used in the donor chamber of the perfusion apparatus. When the vehicle was 20% *w*/*v* HPβCD in saline (Vehicle B) [3,19,35], permeabilities expectedly increased, as cyclodextrins are well-known permeability enhancers and solubilizers utilized to increase ocular bioavailability [39]. The increase was markedly higher for the highly lipophilic parent estrogen (5- to 10-fold) than that of its much more hydrophilic bioprecursor prodrug (2–3-fold) and, therefore, the steady-state transcorneal flux differences between DHED versus E2 decreased to 3–5-fold (Table 1). Using rabbit cornea, we have also established a proportional relationship between J_ss_ and DHED concentrations, as J_ss_ was 14.4 ± 1.0 and 1048 ± 102 pmol·cm^−2^·h^−1^ for substrate concentration of 1 µM and 100 µM DHED, respectively. Importantly, no DHED-to-E2 formation was detected through analysis of cornea homogenate (Figure S1A), while a rapid appearance of E2 from the prodrug could be measured in retina homogenate upon incubation of the prodrug (Figure S1B). This reductive metabolism to E2 was also shown earlier not to be accompanied by hydrogen peroxide formation, as a marker for retinotoxic side-effect [36].

In vivo substantiation of the ex vivo and in vitro findings (Table 1 and Figure S1) was done by instilling eye drops (0.1% *w*/*v* DHED) into the eyes of OVX rats, similarly to our earlier studies [3,19], and following the formation of the parent hormone at the target site (retina) as well as in the blood. As shown in Figure 3, the topically applied prodrug produced the expected rapid formation of E2 in the retina, while this was absent in the blood as DHED remained inert in the circulation. We have also concluded that the retina’s E2 content 24 h after a single DHED eye drop (425 ± 176 pg/g) was not significantly different from that of three-week q.d. treatment upon collecting blood 24 h after the last eye drop administration (389 ± 141pg/g), suggesting lack of retinal hormone accumulation after topical DHED. Importantly, unlike in case of E2 eye drops, typical markers of peripheral estrogenicity [40,41] were also absent, as the uterine and AP wet weights were indistinguishable from those of control group (Figure 4). LC-MS/MS analysis of the blood (Table 2) unequivocally confirmed that no peripheral exposure to the hormone occurred in OVX animals receiving the three-week q.d. DHED eye drops. This further confirmed the distinguishing feature of DHED in terms of CNS-selective bioactivation to E2, that the prodrug remains inert in and is rapidly eliminated from the circulation independently of the route of drug administration [22,23]. Because of the target-specific formation of the neuroprotective hormone, it also was not surprising that identical treatment schedule in an animal model of glaucoma [17] brought about the same extent of neuroprotection (Figure S2) as in our previous studies upon three-week q.d. topical treatments with the hormone itself [3], yet without peripheral liability (Table 2). A follow-up study will specifically be devoted to DHED-based efficacy studies using various animal models of ocular neurodegeneration, as well addressing potential exposure of the brain to E2 from DHED eye drops.

We have also started the initial survey whether we could establish similar formation of E2 in the larger rabbit eye from topical DHED encouraged by the outcomes of corneal transport studies (Table 1). For intact female rabbits, the stable isotope-labeled d_3_-DHED eye drop was used. This also allowed us to simultaneously quantify endogenous E2 in the female rabbit retina (221 ± 40 pg/g) and blood (20 ± 6 pg/mL) and verify whether this treatment triggered or inhibited endogenous E2 formation. Similar to what we have found in the rodent brain [22], we could not distinguish the endogenous E2 contents in the rabbit retina between the treatment and vehicle control groups. This observation confirmed that the inert prodrug did not influence endogenous E2 formation. Therefore, E2 measured in the rabbit retina after topical prodrug originated from the site-specific reductive metabolism of DHED to the parent estrogen (Figure 1). The peak retinal concentration of d_3_-E2 was reached about 1 h after the eye drop treatment (18 ± 7 ng/g), while the maximum prodrug concentration in the blood was measured after about 30 min post-treatment (8 ± 2 ng/mL). Simultaneously, the presence of the hormone in the female rabbit blood was negligible after an initial slight increase at the 15 min time point (18 ± 9 pg/mL). In addition, while we found that 120 ± 28 pg/g d_3_-E2 remained in the retina, it was absent in the blood 24 h post-treatment. A subsequent study will specifically address the impact of chronic topical DHED treatment on these small mammals.

In conclusion, our study presented here shows the premise of the DHED bioprecursor prodrug for topical application to safely and efficaciously deliver E2, a powerful neuroprotectant, into the eye while sparing the rest of the body from unwanted hormonal effect and provides rationale for further investigation about the full potential of our approach for ocular neurotherapy.

## Figures and Tables

**Table 1:** Transcorneal flux (J_ss_, pmol·cm^−2^·h^−1^) of DHED versus E2 at 10 µM substrate concentration at saline (Vehicle A) and 20% *w*/*v* HPβCD in saline (Vehicle B), respectively, in rat, pig, and rabbit corneas ex vivo. Data are given as average ± SD (*n* = 3) ^1^.

Species	Vehicle A		Vehicle B	
	d_3_-E2	DHED	d_3_-E2	DHED
Rat	6.8 ± 1.5	69.0 ± 3.3	76.5 ± 5.4	234.0 ± 11
Rabbit	17.9 ± 1.4	184.3 ± 18.1	89.0 ± 5.5	383.0 ± 5.7
Pig	16.7 ± 1.9	171.0 ± 28.0	105.5 ± 4.3	511.2 ± 64.5

^1^ ANOVA details of this 2 × 2 × 3 factorial experiment were summarized in Table S1.

**Table 2:** Assessment of circulating E2 after three-week q.d. eye drop treatments (0.1% *w*/*v* DHED or E2 in 20% *w*/*v* HPβCD in saline solution) of OVX rats. Blood was collected 24 h after the last treatment. Blood E2 level 24 h after a single DHED eye drop is given as reference. Data are average ± SEM (*n* = 6). * Significant difference compared to control, *p* < 0.05, *n* = 6; ** Significant difference compared to DHED treatments, *p* < 0.05, *n* = 6.

Treatment	Serum E2 (pg/mL)
Vehicle eye drops (control)	8 ± 3
DHED eye drops, single dose	9 ± 4
DHED eye drops 3 weeks, q.d.	8 ± 5
E2 eye drops 3 weeks, q.d.	453 ± 98 *^,^**

## Supplementary Materials

The following are available online at https://www.mdpi.com/1999-4923/12/5/456/s1, Table S1: 2 × 2 × 3 factorial ANOVA of ex vivo steady-state transcorneal fluxes (J_ss_) listed in Table 1; Figure S1: In vitro studies on DHED to E2 metabolism; Figure S2: Neuroprotective effect of DHED-derived E2 on visual function measured as contrast sensitivity at a given spatial frequency in a rat model of glaucoma.
