# Supplementary material for: Retina-Targeted Delivery of 17β-Estradiol by the Topically Applied DHED Prodrug
Source: Pharmaceutics. 2020 May 16;12(5):456. doi: 10.3390/pharmaceutics12050456 (PMC7284430; doi:10.3390/pharmaceutics12050456)
Supplement: Supplementary file 1 [file pharmaceutics-12-00456-s001.pdf]

pharmaceutics

Supplementary Materials: Retina-Targeted Delivery of 17β-Estradiol by the Topically Applied DHED Prodrug

Katalin Prokai-Tatrai, Vien Nguyen, Rebecca Guerra, Daniel L. De La Cruz, Khadiza Zaman, Fatima Rahlouni and Laszlo Prokai

Table S1. 2 × 2 × 3 factorial ANOVA of ex vivo steady-state transcorneal fluxes (J_{ss}) listed in Table 1.

Factor/Interaction	Type III Sum of Squares	Degree(s) of Freedom	F	*p*	η^2
Compound [1]	385,008	1	577	<0.001	0.472
Formulation [2]	217,716	1	326	<0.001	0.267
Species [3]	68,634	2	51.4	<0.001	0.084
Compound × Formulation	56,421	1	84.6	<0.001	0.069
Compound × Species	46,099	2	34.5	<0.001	0.056
Formulation × Species	16,006	2	12.0	<0.001	0.020
Compound × Formulation × Species	10,226	2	7.66	0.003	0.013
Error	16,015	24			0.020

[1] E2 and DHED (2); [2] Without and with HPβCD (2); [3] Rat, rabbit and pig (3).

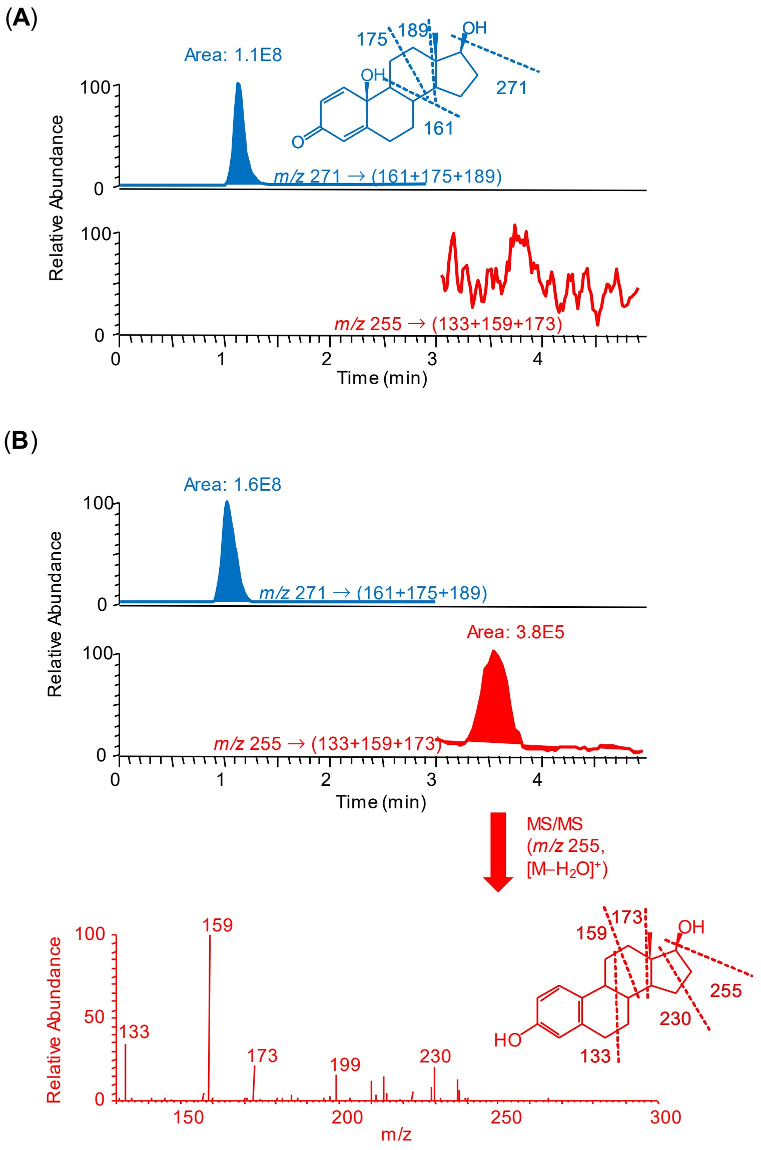

Figure S1. In vitro studies on DHED to E2 metabolism. **(A)**. Evidence for no detectable DHED (blue) to E2 (red) conversion in the acceptor compartment of the diffusion chamber used as established by LC-APCI-MS/MS assay during a 2 h in vitro transport experiment of DHED (10 μM) through pig cornea. Experiments in pig and rat cornea homogenates (20% *w*/*v*) after incubation of DHED (100 nmol/mL) at 37 °C yielded the same results as shown in chart **A**. **(B).** Conversion of DHED (blue) to E2 (red) occurred rapidly in the pig retina with 533 ± 158 pmol·min^{-1} (mg protein)$^{-1}$ (average ± SEM, *n* = 3) initial rate of formation.

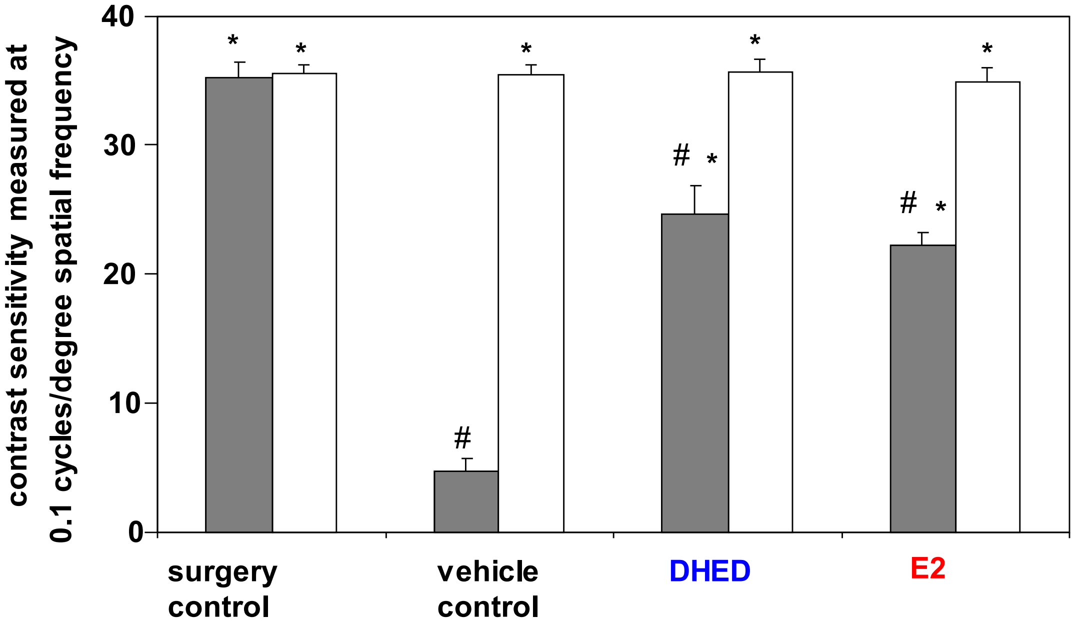

Figure S2. Neuroprotective effect of DHED-derived E2 on visual function measured as contrast sensitivity at a given spatial frequency in a rat model of glaucoma [17]. E2 eye drops (0.1% *w*/*v* in in 20 % HPBC in saline vehicle) [3,19] served as positive control. DHED (0.1% *w*/*v* in 20 % *w*/*v* HPBC in saline vehicle) or 20 % *w*/*v* HPBC in saline vehicle was administered q.d. as eye drops for three weeks, analogously to that of E2 [3,19]. Contrast sensitivity was measured at a given spatial frequency for each treated eye individually in which IOP was increased (approximately by 25% from baseline [3]) and compared to the ipsilateral vehicle treated eye (white) (*; $p < 0.05$, $n = 3$ per group) or to the sham surgery control (#; $p < 0.05$; $n = 3$ per group) [35].
